# A SERS Sensor Prepared via Electrostatic Self-Assembly of Ta_4_C_3_@AgNP Nanocomposites for Detection of Ziram

**DOI:** 10.3390/bios15070426

**Published:** 2025-07-03

**Authors:** Kai Hua, Liang Li, Pei Liang

**Affiliations:** 1 College of Science, China Jiliang University, Hangzhou 310018, China; p22080854007@cjlu.edu.cn; 2 College of Optical and Electronic Technology, China Jiliang University, Hangzhou 310018, China

**Keywords:** MXene nanosheet, AgNPs, SERS, photoinduced charge transfer, Ziram

## Abstract

MXenes are a class of two-dimensional materials exhibiting excellent surface-enhanced Raman scattering (SERS) performance. Currently, the SERS studies of MXenes have been primarily focused on those with M_2_X and M_3_X_2_ structural motifs. In order to expand the SERS sensing application based on MXenes, in this paper, a SERS sensor made of Ta_4_C_3_@AgNP nanocomposite material was fabricated by electrostatic self-assembly. Tests such as different concentrations of R6G probe molecules showed that the minimum detection limit of this SERS sensor was 10^−8^ M, demonstrating excellent sensitivity. When different test areas are selected, the relative error of intensity under the same wave number is less than 10.7%, showing good repeatability and consistency. Furthermore, the Ta_4_C_3_@AgNP nanocomposite SERS sensor was used to detect the pesticide Ziram, and a quantitative model was established. Application detection indicates that this sensor has good sensitivity for the pesticide Ziram, and the minimum detection limit was 10^−6^ M, which exceeded national standard requirements. The findings of this study have potential application value in the fields of food safety and environmental protection.

## 1. Introduction

In recent years, two-dimensional materials have emerged as a frontier in materials science research due to their unique physicochemical properties and extensive application prospects. Following graphene, MXenes have emerged as a new class of two-dimensional transition-metal carbides or nitrides, quickly garnering widespread attention from both the academic and industrial sectors [1]. The MXene materials are derived from the MAX phase, which is obtained through selective etching of the A-layer elements (typically belonging to group III or IV). The general formula for MXenes is M_n+1_X_n_T_X_, where M represents a transition metal, X denotes carbon or nitrogen, and T_X_ refers to a surface termination group such as –OH, –O, or –F [2,3]. The unique structure of MXene materials endows them with exceptional electrical, mechanical, and chemical properties [4,5], thereby demonstrating significant potential for applications in energy storage, catalysis, sensing, and environmental remediation [6,7]. As a two-dimensional non-precious-metal material, an MXene demonstrates remarkable surface-enhanced Raman scattering (SERS) activity. Its distinctive electronic and optical properties, coupled with a high specific surface area, exceptional electrical conductivity, and hydrophilicity [8,9], render it highly suitable as a SERS substrate for the sensitive detection of molecules [10].

At present, MXene-based SERS mainly focuses on M_2_X and M_3_X_2_ structures. Peng et al. [11] first reported that Nb_2_C and Ta_2_C MXenes exhibited significant SERS enhancement. Ta_2_C MXenes can sensitively detect and accurately identify the spike protein of SARS-CoV-2, with a detection limit as low as 5 × 10^−9^ M, which is conducive to achieving real-time monitoring and early warning of the novel coronavirus. Lan et al. [12] proposed a new molecular enrichment method based on vacuum-assisted filtration, achieving ultrafine molecular enrichment. As a result of the adoption of a two-dimensional miniaturization strategy, the V_2_C detection limit of R6G reached 1 × 10^−7^ M. In 2017, the Ti_3_C_2_T_X_ MXene was first demonstrated to have good SERS activity as a SERS substrate; it significantly enhanced the Raman signal that showed R6G [10], and the enhancement factor could reach 10^−6^ through calculation. This indicates that MXenes have the potential for biomedical or environmental applications. By calculating the cohesive and formation energies of Ti_n+1_C_n_, Shein et al. [13] pointed out that the larger the n value, the higher the stability of the nanostructure. In 2024, a high-performance Ti_3_CN was reported. Through two-dimensional morphology regulation and molecular enrichment strategies, the SERS sensitivity of Ti_3_CN MXene was further improved [14]. The Ti_3_CN MXene SERS substrate has also been successfully used for the detection of prohibited fish drugs at low concentrations and a detection limit of 1 × 10^−7^ M for the R6G molecule. In 2024, Lan et al. [15] reported two M_4_X_3_-structured MXenes as SERS substrates for the first time. These MXenes have excellent surface-enhanced Raman scattering capabilities and achieve an enhancement coefficient of approximately 10^5^ times for analytes. However, it can be seen from this study that the sensitivity of Ta_4_C_3_ still needs to be improved. At present, the research on MXenes as SERS substrates is not extensive enough, and the sensitivity of these materials is not high enough. Therefore, precious metals are introduced to enhance the electromagnetic field and improve the SERS sensitivity.

In this work, we successfully synthesized Ta_4_C_3_@AgNP nanocomposites via electrostatic self-assembly. The composition of the nanocomposites was analyzed using X-ray photoelectron spectroscopy (XPS), while their morphology was characterized through scanning electron microscopy (SEM) and transmission electron microscopy (TEM). The SERS properties of the Ta_4_C_3_@AgNP nanocomposite material were investigated using probe molecules, and its enhancement mechanism was explored. Furthermore, it was fabricated into a sensor and applied for the detection of Ziram. The detection demonstrated that Ta_4_C_3_@AgNP nanocomposite material had high sensitivity and repeatability, which proved its promising application in agricultural detection.

## 2. Materials and Methods

### 2.1. Materials

Tantalum aluminum carbide (Ta_4_AlC_3_, 99%) was purchased from Foshan Xinxin Technology Co., Ltd. (Foshan, China). Hydrofluoric acid (HF, 49 wt%) and Ziram (C_6_H_12_N_2_S_4_Zn, 95%) were purchased from Ron. Anhydrous calcium chloride (CaCl_2_, 96%) was purchased from Sinopharm Group Chemical Reagent Co., Ltd. (Shanghai, China). Polydiallyl dimethyl ammonium chloride (PDDA, 20% by weight) was purchased from Yuan Ye Bio (Shanghai, China). Silver nitrate (AgNO_3_, 99%), sodium citrate (C_6_H_5_Na_3_O_7_, 98%), hydroxide tetramethyl ammonium (TMAOH, 25% by weight), and sodium borohydride (NaBH_4_, 96%) were obtained from Aladdin (Shanghai, China). Citric acid (C_6_H_8_O_7_, 99.5%) was sourced from McLin (Shanghai, China). All chemicals did not require further purification processes.

### 2.2. Preparation of AgNPs

The preparation of a silver colloid was carried out using the sodium citrate reduction method [16]. A total of 0.3 mL of AgNO_3_ solution was combined with 10 mL of sodium citrate in a round-bottom flask. Subsequently, 1.8 mL of citric acid was added, and the mixture was allowed to stand at room temperature for 3 min. Under continuous stirring, 0.6 mL of freshly prepared NaBH_4_ was then gradually introduced into the solution, followed by a static period of 5 min. The mixture was then heated to 100 °C and kept in a boiling state for 20 min, followed by cooling in an ice bath.

### 2.3. Synthesis of Ta_4_C_3_@AgNP Nanocomposites

**Figure 1:**
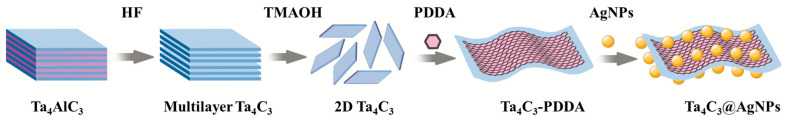
Schematic diagram of the process of preparation for the Ta_4_C_3_@AgNP nanocomposite material.

The preparation process for the Ta_4_C_3_@AgNP nanocomposite material is illustrated in Figure 1. The Ta_4_C_3_ MXene was synthesized by etching Ta_4_AlC_3_ powder in HF to remove Al layers [17,18], washing to neutral pH, intercalating with TMAOH, and finally delaminating via ice-bath ultrasonication to obtain few-layer nanosheets.

The composite material Ta_4_C_3_@AgNPs was synthesized using the method of electrostatic self-assembly [19,20]. To the 10 mL dispersion of Ta_4_C_3_ MXene, 4 mL of PDDA solution was slowly added under continuous stirring for 24 h. Subsequently, the resulting product was centrifuged at 4000 rpm for 1 h. The precipitate obtained was washed with deionized water and subjected to centrifugation three times. Finally, the product was added dropwise to the AgNP solution and stirred for 3 h. The mixture was then centrifuged and washed three times to obtain the Ta_4_C_3_@AgNP nanocomposite material.

### 2.4. Characterization

The XRD patterns of the samples were measured using an X-ray diffractometer (XRD, Rigaku SmartLab, Rigaku Corporation, Tokyo, Japan). The morphological structure of the samples was characterized by scanning electron microscopy (SEM, Sigma 360, Carl Zeiss AG, Oberkochen, Germany), transmission electron microscopy (TEM, JEM-2100F, JEOL Ltd., Tokyo, Japan), and atomic force microscopy (AFM, Dimension Icon, Bruker Corporation, MA, USA). An X-ray photoelectron spectrometer (XPS, Scientific K-Alpha, Thermo Fisher Scientific Inc., MA, USA) was utilized to record the X-ray photoelectron spectra. A zeta potential analyzer (DLS, Zetasizer Pro, Malvern Panalytical Ltd., Worcestershire, UK) was used to measure the zeta potential. Raman measurements were acquired using a Raman spectrometer (Raman, inVia, Renishaw plc, Gloucestershire, UK).

### 2.5. UV-Vis Measurements

First, the liquid sample was diluted to an appropriate concentration, and a solvent that exhibits transparency within the ultraviolet spectrum was selected. Prior to use, the cuvette was thoroughly cleaned to prevent potential contamination. The UV–visible spectrophotometer (UV–Vis, Hitachi UH4150, Hitachi UH4150, Tokyo, Japan) was activated and allowed to preheat for 30 min, and subsequently, the scanning wavelength range was set from 200 nm to 900 nm. Initially, blank correction was conducted using pure solvent, and then the prepared sample was loaded into the cuvette for measurement. Finally, the absorption spectrum obtained was meticulously recorded.

### 2.6. Raman Measurements

The Ta_4_C_3_ MXene dispersion was deposited as droplets on a silicon substrate and allowed to dry naturally. Raman measurements were conducted using a 50× objective lens with an excitation laser wavelength of 532 nm, a laser power of 0.5 mW, an acquisition time of 5 s, and three accumulations while scanning within the range of 1944 to 197 cm^−1^. The Ta_4_C_3_@AgNP nanocomposite was drop-cast onto a silicon wafer and allowed to air dry, forming a uniform film. Both R6G and Ziram were tested in solution form, with their Raman signals acquired by dropwise application onto the SERS substrate. Ziram detection using the Ta_4_C_3_@AgNP nanocomposite was performed with a laser power of 273 mW, an acquisition time of 3 s, and three accumulations. Spectral scanning was conducted over the range of 1800 to 200 cm^−1^.

## 3. Results

### 3.1. Characterization of Ta_4_C_3_@AgNP Nanocomposites

**Figure 2:**
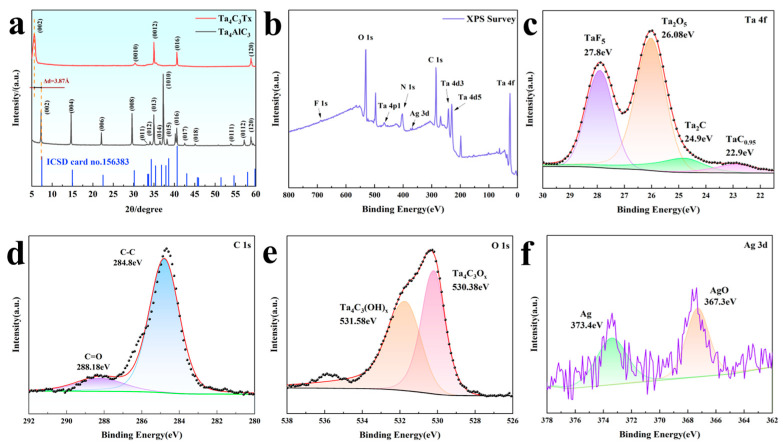
(**a**) XRD patterns of the Ta_4_AlC_3_ MAX phase and Ta_4_C_3_ MXene; (**b**) XPS spectrum of the Ta_4_C_3_@AgNP nanocomposite material; (**c**) Ta 4f XPS spectrum of the Ta_4_C_3_@AgNP nanocomposite material; (**d**) C 1s XPS spectrum of the Ta_4_C_3_@AgNP nanocomposite material; (**e**) O 1s XPS spectrum of the Ta_4_C_3_@AgNP nanocomposite material; (**f**) Ag 3d XPS spectrum of the Ta_4_C_3_@AgNP nanocomposite material.

Figure 2a shows the XRD comparison pattern of the Ta_4_AlC_3_ precursor and the Ta_4_C_3_T_X_ formed after etching. Through the analysis of the diffraction characteristics, the transformation from MAX to MXene can be observed. The Ta_4_AlC_3_ precursor presents a typical hexagonal crystal system MAX phase structure, with the main characteristic peaks located at 7.2°, 14.6°, 22°, 29.6° and 37.2°, corresponding to the (002), (004), (006), (008) and (1010) crystal planes, respectively (ICSD card 156383). After HF acid etching treatment, the most significant change was that the peak (002) shifted from 7.2° to a lower angle of 5.6°, indicating that the lattice parameter of A significantly expanded from the initial value of 23.71 A to 31.45 A, with an increase of 32.6%. This lattice expansion is a direct result of the selective etching of Al atomic layers and the insertion of surface terminating groups (such as –O, –OH, –F, etc.) and water molecules between the layers. Meanwhile, the peaks at 30.4° and 35.1° originate from the diffraction of the (0010) and (0012) planes of Ta_4_C_3_, indicating the successful preparation of MXene. The broadening of XRD peaks and the reduction in intensity indicate a decrease in order degree, which is consistent with the characteristics of two-dimensional materials [21,22].

Due to the presence of abundant functional groups on the surface of the Ta_4_C_3_ MXene, it exhibits a negatively charged characteristic, with a measured Zeta potential of −42.78 mV. AgNPs were synthesized by reducing sodium citrate, resulting in the presence of citrate ions on their surface, which imparts a negative charge to AgNPs. Consequently, PDDA, as a positively charged modifying agent, was employed to connect two negatively charged materials. A simple electrostatic self-assembly method was utilized to prepare the Ta_4_C_3_@AgNP nanocomposite material.

The full-spectrum XPS information of the prepared Ta_4_C_3_@AgNP nanocomposite material is presented in Figure 2b. The fitting peaks for Ta 4f, as shown in Figure 2c, correspond to TaF_5_, Ta_2_O_5_, Ta_2_C, and TaC_0.95_, with binding energies of 27.8 eV, 26.08 eV, 24.9 eV, and 22.9 eV [23,24], respectively. The peaks in the C 1s spectrum of sample C, as shown in Figure 2d, are located at 284.8 eV and 288.18 eV, corresponding to C-C and C=O bonds, respectively. As illustrated in Figure 2e, the spectra of the O 1s region can be attributed to Ta_4_C_3_O_X_ and Ta_4_C_3_(OH)_X_, with binding energies of 530.38 eV and 531.58 eV corresponding to Ta_4_C_3_O_X_ and Ta_4_C_3_(OH)_X_. The spectrum peaks in the Ag 3d region, as shown in Figure 2f, are located at 373.4 eV and 367.3 eV, corresponding to Ag and AgO, respectively. The XPS spectrum indicates that Ta, C, O, and Ag are the predominant elements present, and no characteristic peaks associated with Al were detected. This confirms that the Al atomic layer has been successfully removed through etching. These results demonstrate that Ag nanoparticles have been successfully incorporated into Ta_4_C_3_ nanosheets.

**Figure 3:**
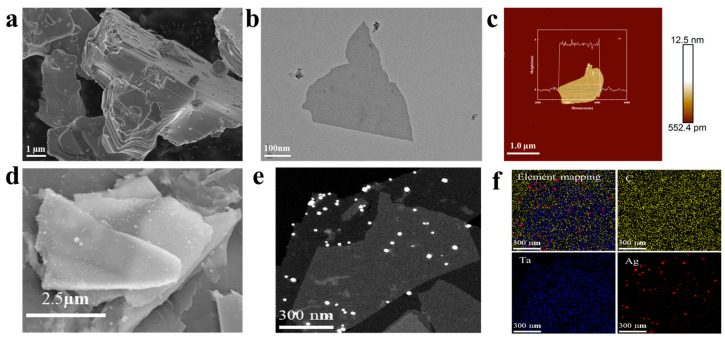
(**a**) SEM image of the Ta_4_AlC_3_ MAX phase; (**b**) TEM image of 2D Ta_4_C_3_ MXene; (**c**) AFM image of 2D Ta_4_C_3_ MXene. (**d**) SEM images of Ta_4_C_3_@AgNPs; (**e**) TEM image of Ta_4_C_3_@AgNPs; (**f**) elemental mapping images of Ta_4_C_3_@AgNPs.

As shown in Figure 3a, the Ta_4_AlC_3_ MAX phase exhibits a layered particle morphology prior to etching. The TEM image of the sample prepared from Figure 3b indicates that the material exhibits a distinct layered structure. As shown in Figure 3c, AFM measurements reveal that the thickness of the few-layer nanosheets is approximately 4.56 nm. These results of characterization collectively demonstrate that Ta_4_C_3_ nanosheets have been successfully synthesized. The results of the SEM and EDS analysis for the prepared samples are presented in Figure 3d. With elements Ta, Ag, and C of the prepared samples being shown, AgNPs can be observed to be distributed on nanosheets. Figure 3e presents the TEM micrograph of the Ta_4_C_3_@AgNP nanocomposite material. As observed, the Ag nanoparticles exhibit an average particle size of approximately 40 nm and are uniformly distributed across the MXene substrate with no signs of agglomeration. Figure 3f shows the elemental mapping of Ta_4_C_3_@AgNP nanocomposites. The element mapping images of Ag, Ta, C, and their superpositions describe the distribution of Ag, Ta, and C. The presence of Ag and Ta further indicates that Ag is adsorbed on the surface of the MXene. This indicates that we have successfully synthesized high-quality Ta_4_C_3_@AgNP nanocomposite material.

### 3.2. SERS Performance of Ta_4_C_3_@AgNP Nanocomposites

**Figure 4:**
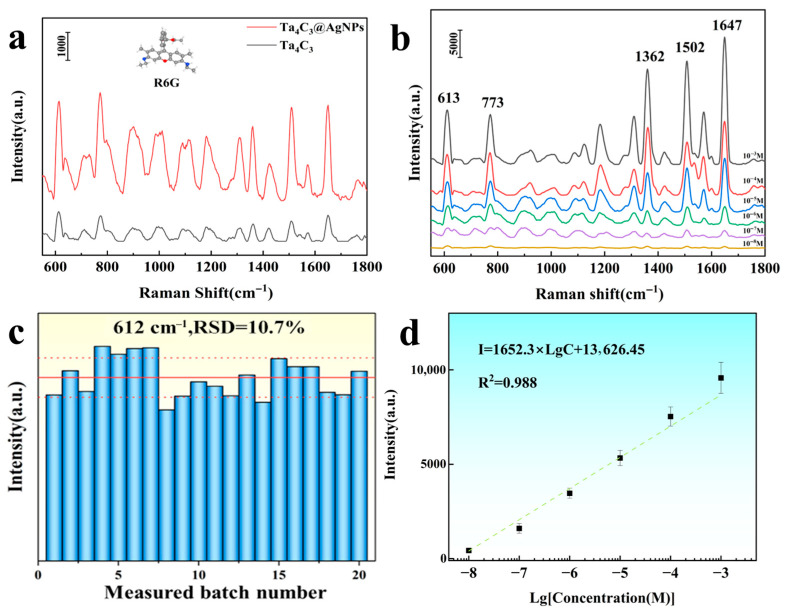
(**a**) The Raman spectra of R6G molecules (1 × 10^−6^ M) adsorbed on Ta_4_C_3_ and Ta_4_C_3_@AgNPs; (**b**) the Raman spectra of R6G adsorbed on Ta_4_C_3_@AgNPs at different concentrations ranging from 10^−3^ to 10^−8^ M; (**c**) statistical distribution of peak intensity at 612 cm^−1^; (**d**) the relationship between the Raman intensity at a shift of 612 cm^−1^ and the logarithm of R6G concentration.

The commonly used organic dye molecule Rhodamine 6G (R6G) was selected as a probe molecule to investigate the SERS performance of the Ta_4_C_3_ MXene and Ta_4_C_3_@AgNP nanocomposite material. As shown in Figure 4a. The detection of R6G molecules indicated that the detection effect of Ta_4_C_3_@AgNPs was significantly better than that of bare Ta_4_C_3_. This enhancement is primarily attributed to the LSPR of AgNPs, which induces a strong EM field. Each Ag nanoparticle generates an intense local field on its surface, particularly at the edges, thereby amplifying the Raman scattering of nearby molecules. Notably, even sparsely distributed AgNPs can produce substantial SERS signals when incorporated into MXene-based hybrids. For instance, Yang et al. [25] reported that even a few AgNPs on Ti_3_C_2_T_X_ can form “nanoscale gaps” with probe molecules, leading to excellent SERS performance. As long as the analyte resides near the AgNPs or at the AgNP/MXene interface, its Raman signal is markedly enhanced.

As shown in Figure 4b, SERS tests were carried out on the modified samples using a 532 nm laser. The characteristic Raman peaks of R6G at 612 cm^−1^, 773 cm^−1^, 1360 cm^−1^, and 1650 cm^−1^ are clearly observable. The peaks at 612 cm^−1^ and 773 cm^−1^ are attributed to the in-plane C−C−C bending vibrations and out-of-plane C−H bending vibrations within the xylose acid backbone, respectively. In contrast, the peaks at 1360 cm^−1^ and 1650 cm^−1^ correspond to the C−C stretching vibrations of the aromatic ring [26,27]. As the concentration of R6G decreases, the intensity of the characteristic Raman peaks also decreases, with a detection limit reaching as low as 1 × 10^−8^ M. The Raman enhancement factor (EF) serves as a crucial indicator for assessing the enhancement effect of substrates, and its calculation formula is given by [15]

(1) $$EF=\frac{I_{SERS}}{I_{Raman}}\times \frac{N_{Raman}}{N_{SERS}}$$

The intensity of the characteristic Raman peak at 612 cm^−1^ was selected to calculate the enhancement factor. The calculated Raman enhancement factor for the Ta_4_C_3_@AgNP nanocomposite material is found to be 1.53 × 10^6^. The working curve depicted in Figure 4b illustrates the relationship between Raman intensity (I) and R6G concentration (c) on a logarithmic scale. As shown in Figure 4d, a strong linear correlation is observed within the concentration range of 1 × 10^−3^ to 1 × 10^−8^ M, with a correlation coefficient (R^2^) of 0.988. The fitting equation is expressed as Lg I = 1652.3 Lg C + 13,626.45. The aforementioned results indicate that the Ta_4_C_3_@AgNP nanocomposite material exhibits excellent SERS activity, suggesting its potential for quantitative analysis. The consistency of the SERS signals from the samples was tested by randomly selecting 20 points to record the results, as shown in Figure 4c. The relative standard deviation (RSD) calculated for the characteristic Raman peak at 612 cm^−1^ was found to be 10.7%. These findings demonstrate that the Ta_4_C_3_@AgNP nanocomposite material exhibits excellent consistency in the SERS signal response. For the substrates prepared in three independent batches, when detecting the same concentration of R6G, the relative standard deviation of the main Raman peak remained below 12%, indicating good consistency between batches and reliable repeatability. The aforementioned results indicate that Ta_4_C_3_@AgNP nanocomposite material is an excellent substrate material for SERS, suggesting its potential for quantitative analysis.

**Figure 5:**
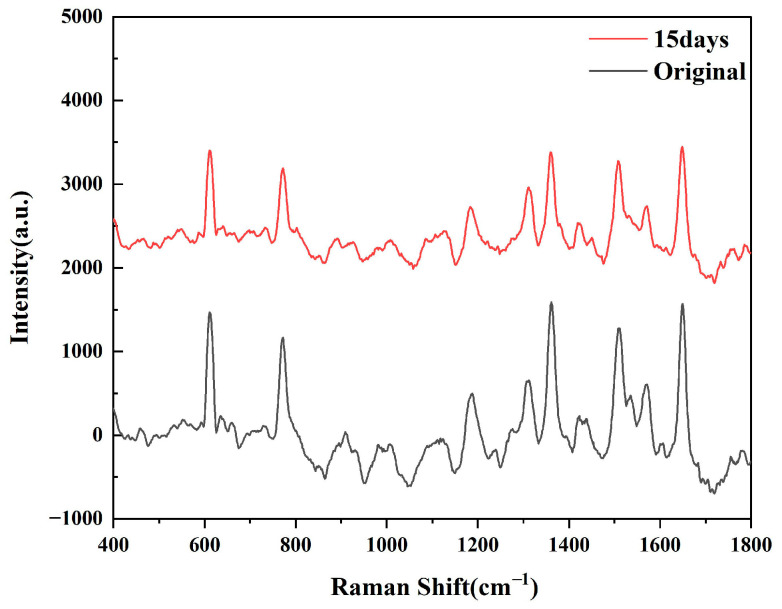
SERS spectra produced by R6G on Ta_4_C_3_@AgNPs 15 days later.

As shown in Figure 5, after testing the SERS spectra generated by R6G at different times, we can see that the intensity of the characteristic peaks of R6G has decreased, but there is no sharp fluctuation. Based on the original spectral data, the calculation results show that the sensor still retains approximately 70.6% of the original Raman intensity after 15 days, which proves that the substrate has time stability.

**Figure 6:**
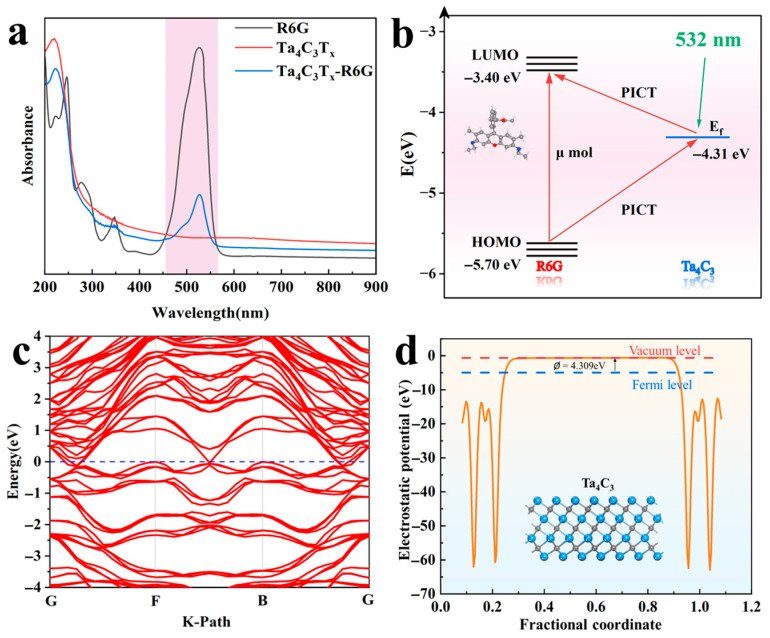
(**a**) UV-vis absorption spectra of R6G, Ta_4_C_3_, and the Ta_4_C_3_-R6G composite; (**b**) diagram of the energy level and charge transfer pathways in the Ta_4_C_3_-R6G system; (**c**) electronic band structure of monolayer Ta_4_C_3_; (**d**) work function of the Ta_4_C_3_ monolayer.

A theoretical and experimental investigation of the Raman enhancement mechanism for the Ta_4_C_3_ MXene has been carried out. The ultraviolet–visible absorption spectrum of Ta_4_C_3_ MXene is shown in Figure 6a. It can be observed that the MXene exhibits no absorption bands within the visible-light range, indicating a lack of potential for electromagnetic field enhancement. Furthermore, at approximately 527 nm, photoluminescence (PL) quenching of R6G is observed after its adsorption onto the Ta_4_C_3_ MXene, suggesting the occurrence of strong interfacial charge transfer (CT) between them [14]. Therefore, the Raman enhancement mechanism of the Ta_4_C_3_ MXene is primarily attributed to CM [15]. As illustrated in Figure 6c,d, the electronic band structure, density of states (DOS), and work function of Ta_4_C_3_ were obtained through calculations of density functional theory (DFT). The electronic bands of Ta_4_C_3_ intersect multiple Fermi levels, demonstrating its metallic properties.

According to the Golden Fermi rule, the probability of electronic transition $\omega_{IK}$ is given by [14]

(2) $$\omega_{IK}=\frac{2\pi }{\hbar }g\left(E_{K}\right){\left|H_{kI}^{\prime }\right|}^{2}$$

The symbol $g\left(E_{K}\right)$ represents the number of available final states within a unit energy range. The magnitude of the density of states influences the transition probability, while the perturbation Hamiltonian $H_{kI}^{\prime }$ denotes the matrix elements between the initial and final states. As shown in Figure 6d, the DOS value of Ta_4_C_3_ at the Fermi level is 4.438 states/eV. The relatively high DOS value facilitates an increase in the probability of electronic transitions, thereby enhancing the SERS performance of MXene substrates. The calculated work function is 4.309 eV. The Fermi level of Ta_4_C_3_ is located at −4.309 eV relative to the vacuum level. For the R6G molecule, the energy levels of the highest occupied molecular orbital (HOMO) and the lowest unoccupied molecular orbital (LUMO) are −5.70 eV and −3.40 eV. The polarization tensor α provides a framework for understanding the mechanisms of molecular interactions with their environment. Specifically, the polarization tensor can be expressed as [12] α=A+B+C; A primarily reflects the inherent Raman activity of the molecule, while B and C illustrate the additional enhancement effects on Raman scattering due to CT processes induced by light between the substrate and the molecule, as well as from the molecule back to the substrate. The energy level schematic of the Ta_4_C_3_-R6G system is illustrated in Figure 6b. The Fermi energy level of the substrate matches with the energy states of the LUMO and HOMO energy levels of the molecule, thereby allowing electrons to transfer between them. Under the illumination of a laser with a wavelength of 532 nm (2.33 eV), both the CT process from the HOMO of R6G to the Fermi level of Ta_4_C_3_ and the CT process from the Fermi level of Ta_4_C_3_ to the LUMO of R6G contribute significantly to Raman enhancement. Furthermore, the excitation wavelength energy is very close to the electronic transition energy of R6G. According to the Herzberg–Teller theory [28], this proximity will further amplify the polarization tensor of the molecule, thus enhancing the Raman signal from R6G molecules. The aforementioned results indicate that the SERS signal from molecules adsorbed on MXene originates from the interface PICT mechanism between MXene and the target molecules.

When the analyte is in contact with both AgNPs and the MXene, it benefits from the synergistic effects of both EM and CT mechanisms. Wang et al. [29] explicitly attributed the improved sensitivity of AuNSs@Ta_4_C_3_T_X_ composites to the enhanced electromagnetic properties of the hybrid substrate. Similarly, Satheeshkumar et al. [30] reported an exceptionally high enhancement factor for MXene–Ag substrates due to the plasmonic synergy between Ag and an MXene. Consistent with these findings, our results demonstrate that the AgNP-functionalized MXene yields significantly stronger SERS signals compared to the pristine MXene, even under low Ag coverage, confirming the presence of a cooperative EM + CT mechanism [31,32,33]. This dual enhancement strategy accounts for the pronounced SERS performance observed in our system.

The theoretical basis for the test results can be summarized as a synergistic effect between electromagnetic field enhancement and charge transfer enhancement: (1) uniformly distributed AgNPs on the surface of the MXene provide a stable localized electric field enhancement, while the conductive properties of the MXene further enhance the localized field effect of metal particles; (2) the enhanced field effect and increased specific surface area facilitate molecular adsorption and charge transfer processes. Together, these factors contribute to the amplification of SERS signals.

### 3.3. Detection of Ziram Using Ta_4_C_3_@AgNP Nanocomposite Sensor

**Figure 7:**
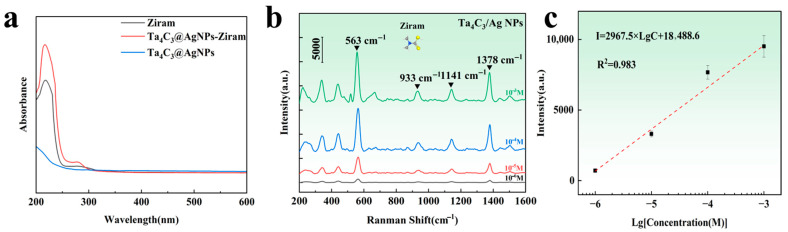
(**a**) UV-Vis absorption spectra of Ziram, Ta_4_C_3_@AgNPs and Ta_4_C_3_@AgNPs–Ziram; (**b**) Raman spectra of different concentrations of Ziram on the Ta_4_C_3_@AgNP nanocomposite material; (**c**) relationship between the logarithm of Raman intensity at a Raman shift of 563 cm^−1^ and the logarithm of Ziram concentration.

The commonly used pesticide Ziram was selected as the target analyte for practical detection. As shown in Figure 7a, the UV-vis absorption spectra of Ziram, Ta_4_C_3_@AgNPs, and Ta_4_C_3_@AgNPs–Ziram exhibit enhanced absorption in the range of 200–300 nm. This enhancement indicates the superposition of characteristic peaks of Ziram, confirming its successful modification onto the surface of the composite material. In the range of 200–256 nm, the absorption peak broadens, indicating that the interaction between the composite material and Ziram may be attributed to chemical adsorption. This result also confirms that the substrate material may exhibit chemical enhancement of the SERS signals for target molecules. The Raman spectrum of the target molecule is illustrated in Figure 7b. It can be observed that the intensity of the Raman peaks decreases with a reduction in Ziram concentration. Characteristic peaks appear near the Raman shifts at 430, 563, 933, 1141, and 1378 cm^−1^. The main Raman peaks of Ziram and the distribution of vibration modes are shown in Table 1. The peak at 430 cm^−1^ corresponds to the stretching vibration mode of the C=S bond, which is a characteristic signal of dithiocarbamate pesticides such as Ziram. The peak at 563 cm^−1^ is attributed to the vibration mode of the S=S, indicating the presence of typical disulfide structures in the molecule. The peak at 933 cm^−1^ corresponds to a coupling mode between C=S stretching vibrations [34]. The 1141 cm^−1^ peak originates from the stretching vibration of the C–N bond and the rocking mode of CH_3_ groups, which are important structural features of carbamate-type molecules. The peak at 1378 cm^−1^ is associated with the stretching vibration of the C–N bond combined with the vibrational contribution from methyl groups, further confirming the presence of nitrogen-containing organic compounds [35].

Furthermore, it is noted that the intensity of these Raman spectral peaks exhibits a linear relationship with the logarithm of the Ziram concentration. As shown in Figure 7c, the linear range extends from 10^−3^ M to 10^−6^ M, with a detection limit that is lower than the national standard [36]. The intensity of the characteristic Raman peak at 563 cm^−1^ was selected to calculate the enhancement factor. The calculated Raman enhancement factor for the Ta_4_C_3_@AgNP nanocomposite material is found to be 1.35 × 10^4^. The linear equation is expressed as I = 2967.5 Lg C + 18,488.6, and the correlation coefficient R^2^ is 0.983. This indicates that a good logarithmic linear relationship has been achieved between SERS intensity and concentration, facilitating both qualitative and quantitative detection of the target substance.

The Ta_4_C_3_@AgNPs MXene nanocomposite material, as a novel detection platform, has broad application prospects in fields such as food safety, environmental monitoring, and water quality assessment. In the future, we will further optimize the structure or surface chemistry of the Ta_4_C_3_@AgNPs MXene nanocomposite to improve its SERS performance.

## 4. Conclusions

In summary, a Ta_4_C_3_@AgNP nanocomposite was successfully fabricated via self-assembly, and molecular detection studies demonstrated its excellent sensitivity and reproducibility. Using first-principles calculations, we demonstrated that Ta_4_C_3_ exhibits excellent electrical conductivity, which enhances charge transfer and further enhances the Raman signal of the Ta_4_C_3_@AgNP nanocomposite. Through practical detection of the pesticide Ziram, the Ta_4_C_3_@AgNP nanocomposite sensor demonstrated excellent sensitivity, achieving an enhancement factor of 1.35 × 10^4^ and exhibiting strong detection capability. The implementation of trace detection for agricultural residues has significant potential to address the food safety issues associated with pesticide residues.

## Figures and Tables

**Table 1:** The main Raman peaks for Ziram and vibration mode assignments.

SERS Spectrum (cm^−1^)	Assignment
430	C=S vibration mode
563	Vibration mode of S=S
933	C=S vibration mode
1141	CH_3_ deformation and CN stretching
1378	CN stretching and CH_3_ rocking

## Data Availability

Data are contained within the article.
